# The Acromioclavicular‐Distal Clavicle 3‐Dimensional All‐Suture Stabilization Technique Without Need for Fluoroscopy and Without Metallic Hardware

**DOI:** 10.1002/atn2.70185

**Published:** 2026-06-22

**Authors:** Marouane Bouloudhnine

**Affiliations:** ^1^ Hand to Shoulder Center, Dr Sulaiman Al Habib Hospital Palm Jumheira, Dubai United Arab Emirates

## Abstract

The acromioclavicular joint's intricate biomechanics and anatomy pose significant challenges in its repair. The “Acromioclavicular‐Distal Clavicle 3‐Dimensional All‐Suture Stabilization Technique” uses unicortical self‐punching all‐suture anchors, which reduce nerve damage risks. This technique emphasizes three core objectives by making the surgical process safe and efficient.

## Graphical abstract

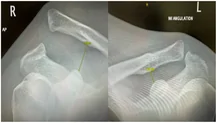

**VIDEO 1:** Arthroscopic demonstration of the all‐suture acromioclavicular joint stabilization technique performed without fluoroscopy or metallic hardware. The patient is positioned in the beach‐chair position. The procedure is performed on the affected shoulder through an arthroscopic viewing portal. Video content can be viewed at https://doi.org/10.1002/atn2.70185.

The comprehension of the biomechanics behind acromioclavicular joint injuries has advanced. It has become evident that restoring both the acromioclavicular and coracoclavicular ligaments is crucial, as the acromioclavicular joint involves more than just the joint between the distal clavicle and the acromion.^1^, ^2^, ^3^, ^4^, ^5^

This technical note describes the “Acromioclavicular‐Distal Clavicle 3‐Dimensional All‐Suture Stabilization Technique” (a modification of the current coracoclavicular tunnel convergence technique) to address the challenges discussed above by simplifying the procedure, using unicortical, self‐punching all‐suture anchors, and avoiding metallic hardware. This technique reduces the risk of nerve or hardware issues and is safe, efficient, and cost‐effective.

## SURGICAL TECHNIQUE

The surgical technique is shown in Video 1 and also illustrated in Figures 1, 2, 3, 4, 5, 6, 7, 8, 9. Figure 8 represents the final paradigm. Figures 10 and 11 show the pre‐ and the postoperative radiograph. Informed consent in detail was obtained for the procedure described.

**FIGURE 1:**
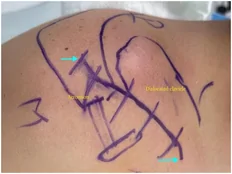
Surgical site preparation and skin incision marking in the right shoulder with the patient positioned in the beach‐chair position. Superolateral view showing the planned skin incision marked with a dermographic pen, with the acromion and dislocated clavicle identified.

**FIGURE 2:**
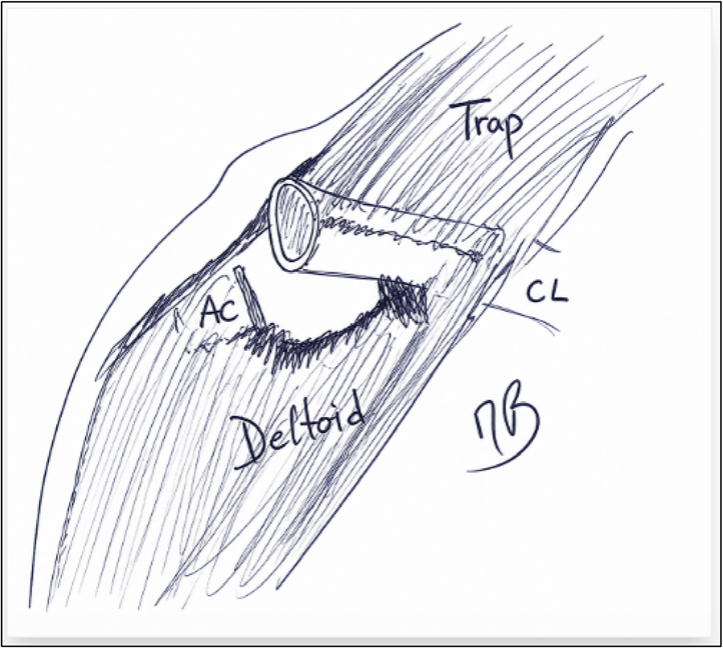
Intraoperative illustration of soft tissue disruption in a stage IIIB acromioclavicular joint dislocation of the right shoulder with the patient positioned in the beach‐chair position. Superior view showing partial detachment of the deltoid muscle, complete dislocation of the acromioclavicular joint, and partial detachment of the trapezius muscle at its clavicular insertion.

**FIGURE 3:**
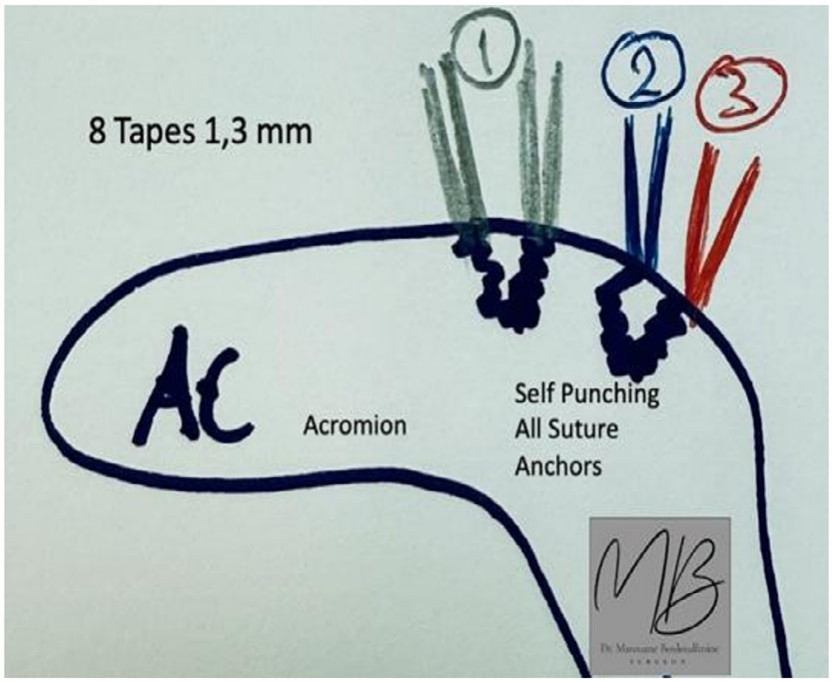
Illustration of all‐suture self‐punching anchor placement for acromioclavicular joint stabilization in the right shoulder with the patient positioned in the beach‐chair position. Superior view showing three anchors placed at the coracoid base/acromion region, with eight 1.3‐mm suture tapes prepared for reconstruction.

**FIGURE 4:**
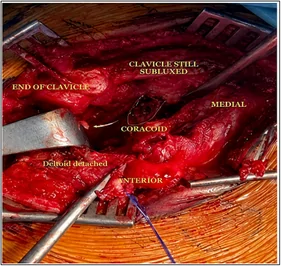
Positioning of all‐suture self‐punching anchors at the coracoid base in the right shoulder with the patient positioned in the beach‐chair position. Intraoperative superior view showing direct visualization of medial‐to‐lateral anchor placement on the coracoid, without penetration of the inferior cortex. A 2‐mm predrill is used in hard bone to prevent slippage.

**FIGURE 5:**
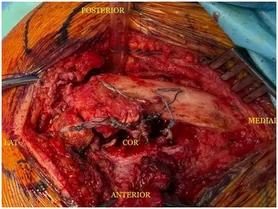
Drilling of bone tunnels in the clavicle and acromion in the right shoulder with the patient positioned in the beach‐chair position. Intraoperative superior view showing preparation of four tunnels using a 2.5‐mm drill bit: two medial vertical tunnels and one lateral vertical tunnel in the clavicle, one horizontal tunnel at the end of the clavicle, and one oblique tunnel in the acromion to allow passage of suture limbs for coracoclavicular and acromioclavicular ligament reconstruction.

**FIGURE 6:**
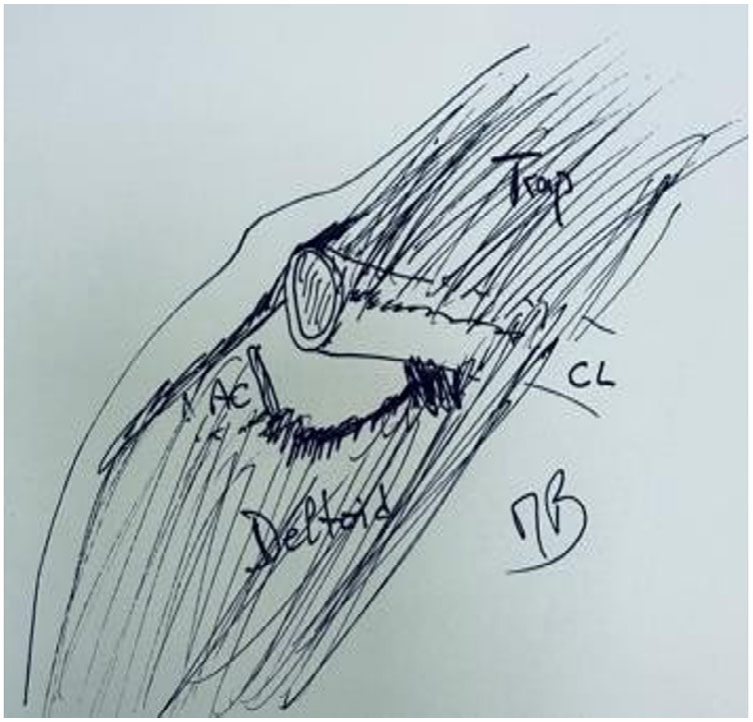
Step‐by‐step suture tape passage for coracoclavicular and acromioclavicular ligament reconstruction in the right shoulder with the patient positioned in the beach‐chair position. Superior‐view illustration showing sequential passage of the suture tapes; medially, two suture tapes traverse from inferior to superior through the vertical clavicular tunnels and are crossed to reconstruct the coracoclavicular ligaments.

**FIGURE 7:**
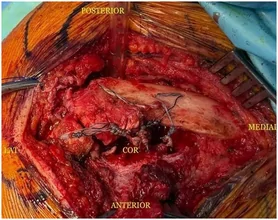
Lateral view of the right shoulder with the patient positioned in the beach‐chair position. Intraoperative view showing the correctly oriented clavicle after preparation for acromioclavicular and coracoclavicular ligament reconstruction.

**FIGURE 8:**
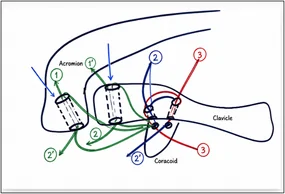
Verification of acromioclavicular joint reducibility after tunnel drilling in the right shoulder with the patient positioned in the beach‐chair position. Superior‐view illustration showing completed reconstruction with anatomic alignment of the acromioclavicular joint and secure suture tape configuration to maintain reduction.

**FIGURE 9:**
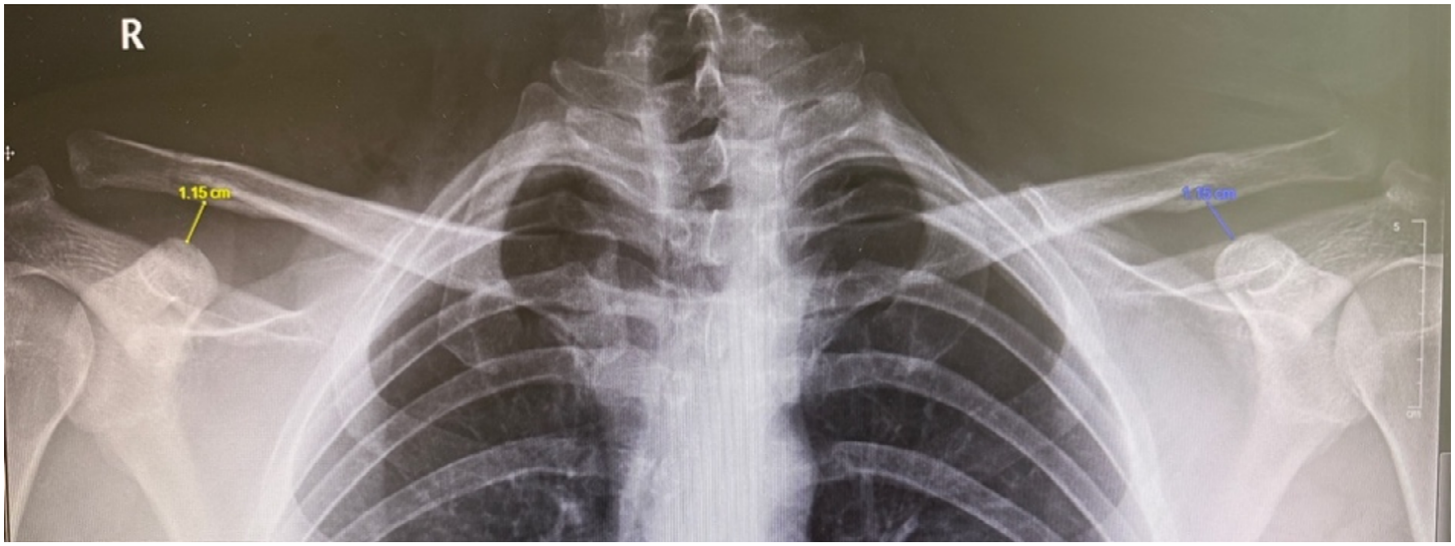
Postoperative anteroposterior radiograph of the right shoulder following acromioclavicular and coracoclavicular ligament reconstruction using the all‐suture technique. The final construct shows reduction and alignment of the acromioclavicular joint without metallic hardware.

**FIGURE 10:**
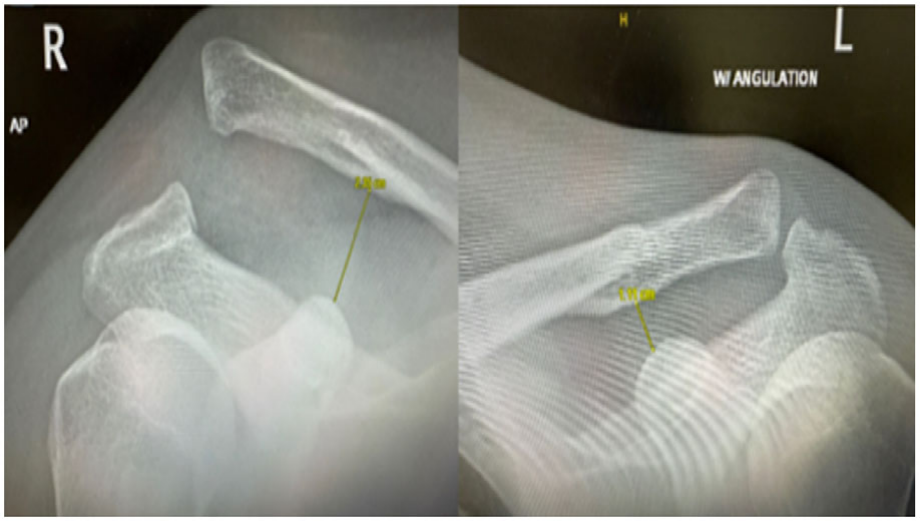
Upright anteroposterior radiographs comparing the affected and contralateral shoulders. The right shoulder shows increased coracoclavicular distance of 2.35 cm compared with 1.15 cm on the left shoulder, indicating marked acromioclavicular joint separation.

**FIGURE 11:**
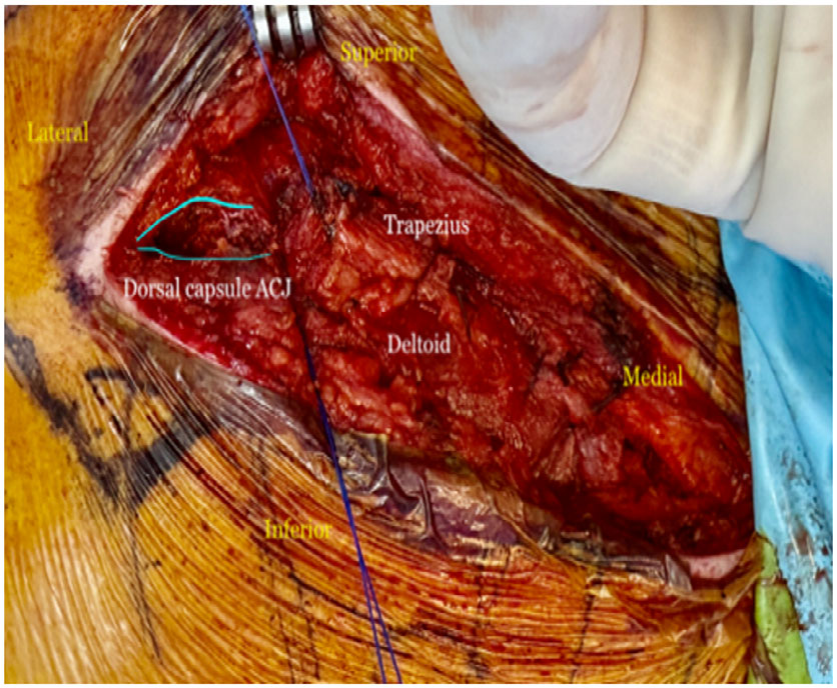
Closure of the deltotrapezial fascia and acromioclavicular joint capsule in the right shoulder with the patient positioned in the beach‐chair position. Intraoperative view showing approximation of the deltotrapezial fascia and dorsal acromioclavicular capsule with nonabsorbable sutures to reinforce the repair and maintain acromioclavicular joint stability.

### Patient Preparation

**FIGURE 12:**
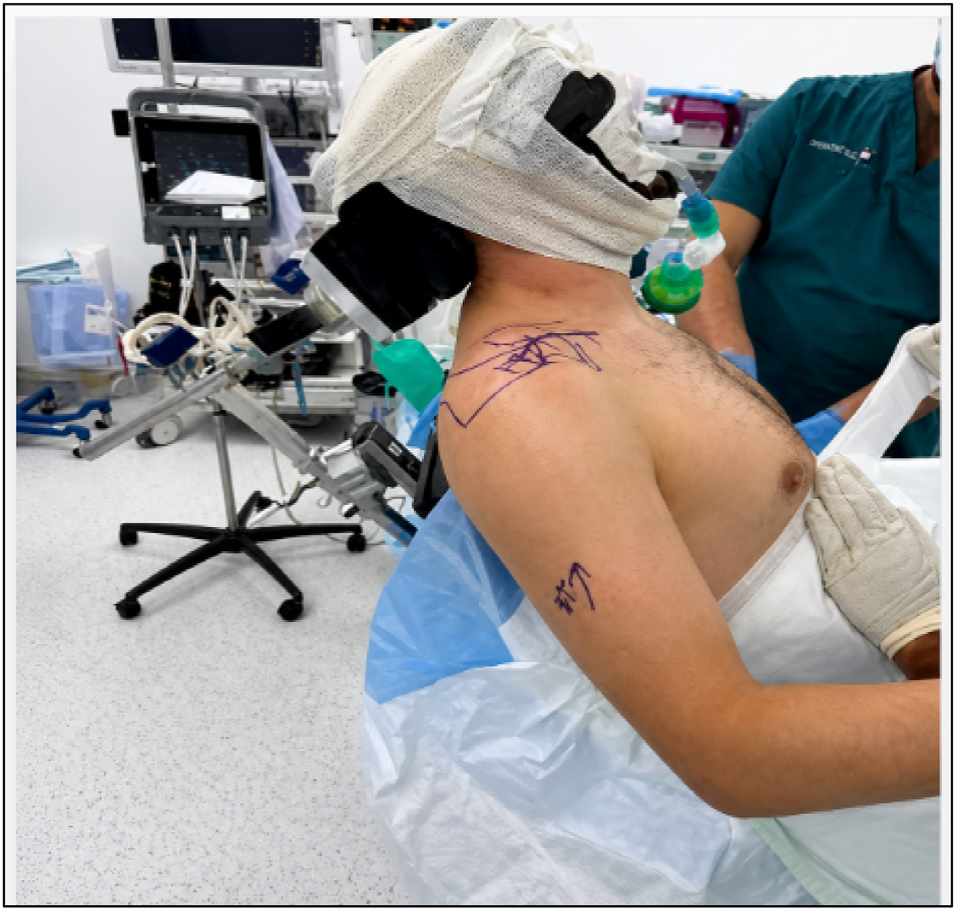
Preoperative setup of the patient in the beach‐chair position for right shoulder surgery. The right upper limb is positioned at the side in neutral flexion to allow anterior operative exposure of the clavicle, coracoid, and acromioclavicular joint.

The patient is placed on beach‐chair position on a surgical table with a removable shoulder support (Figure 12). No fluoroscopy is used.

### Surgical Approach

A 10‐cm longitudinal skin incision is made, initiating medially and then extending it laterally over the third lateral of the clavicle, crossing the acromioclavicular joint (ACJ) and ending at the anterior border of the acromion (Figure 1). The lateral clavicle part of the deltoid is found detached; however, the trapezius is still attached (Figure 2). The coracoid process and remnants of the coracoclavicular ligaments are identified and palpated anteriorly to the distal third of the clavicle, with minimal dissection in this area to preserve the remaining torn ligaments (Figure 3).

### Coracoclavicular Preparation

This step is performed prior to the ACJ reduction as the clavicle's posterior displacement aids the approach of the process of the coracoid as well as the anchors’ placement (Table 1). The lateral one is at 2.5 cm from the ACJ, and the medial one is at 4 cm. A minimum distance of 15 mm needs to be maintained between both to avoid clavicle fracture (Figure 5).

**TABLE 1:** Pearls and Pitfalls of the “AC‐DC 3D All Suture” Technique

Pearls	Pitfalls
• Identify and reference the deltotrapezial fascia, if possible	• Make sure to visualize the entire coracoid (base, lateral, and medial parts)
• Carefully dissect the soft tissues around the lateral clavicle to facilitate ACJ reduction and placement of tunnels	• Make sure to predrill the coracoid entry points of the anchors

AC‐DC 3D, Acromioclavicular‐Distal Clavicle 3‐Dimensional; ACJ, acromioclavicular joint.

### Coracoacromioclavicular Complex Repair

It is done in two separate steps (Figure 6):

1. Coracoclavicular repair is based on the same *medial anchor* (2 tapes, 4 limbs).
2. Acromioclavicular repair is based on the same *lateral anchor* (2 tapes, 4 limbs).

### Joint Reduction and Stabilization

The arm is placed in the position of a “shoulder shrug” to help with the reduction of the ACJ with minimal tension. After checking the reducibility of the dislocation (Figure 7), a temporary k‐wire is used to maintain the reduction.

### Postoperative Management

**FIGURE 13:**
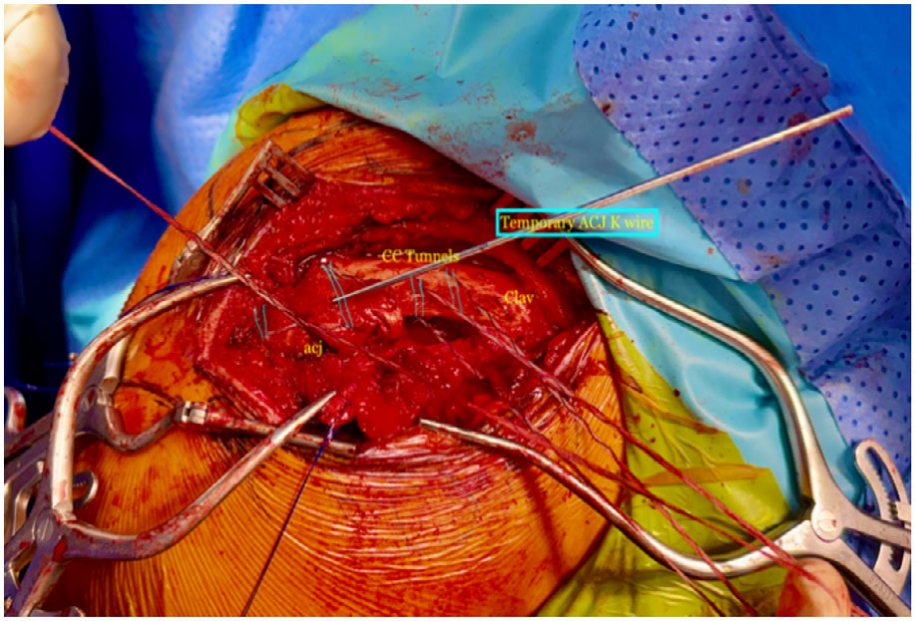
Intraoperative view of the right shoulder with the patient positioned in the beach‐chair position. The image shows the final all‐suture acromioclavicular and coracoclavicular ligament reconstruction construct with the clavicle reduced and secured, confirming stable acromioclavicular joint alignment without metallic hardware.

The deltotrapezial fascia is closed strongly using Mason‐Allen sutures (Figure 9). The postoperative radiograph can show the stability of the repair (Figure 11) and the anatomical restoration of the coracoclavicular distance. The postoperative follow‐up radiograph further confirms maintenance of ACJ reduction and restoration of the coracoclavicular distance (Figure 13).

## DISCUSSION

Several techniques have been proposed to restabilize the ACJ following grade IIIB‐V acute dislocations, with an historical high grade of reduction subsidence and constructs failure.^6^, ^7^

There are still several limitations that must be acknowledged before implementing this technique (Table 2):

1. Surgeons unaware of self‐punching all‐sutures anchor placement methods may experience a learning curve.

**TABLE 2:** Advantages of the “AC‐DC 3D All Suture ACJ” Stabilization Technique

Advantages
• The AC‐DC 3D ALL Suture ACJ Stabilization is a true “all‐suture technique” with no use of metallic hardware such as endobutton or cortical button (cost saving, time saving, potential complication avoided)
• Self‐punching anchors avoid the predrill needed for regular anchor and the difficulty to find the correct hole (quicker procedure)

Disadvantage
• Technique may require a learning curve for surgeons unfamiliar with all‐suture systems
• Visualization and access to the coracoid base may be limited in patients with complex anatomy

AC‐DC 3D, Acromioclavicular‐Distal Clavicle 3‐Dimensional; ACJ, acromioclavicular joint.

Video‐Assisted Minimally Invasive II surgeons should expect a remarkable learning curve when mastering this modern technique because they may not be familiar with the placement techniques of self‐punching all‐suture anchors. Unicortical self‐punching all‐suture anchors not only fixate differently from conventional metallic or screw‐based systems but require an understanding of anatomical landmarks, insertion angles, and tensioning techniques to ensure proper anchor deployment and joint stability. Thus, the high degree of precision and familiarity with arthroscopic tools and navigation required by this advancement in minimally invasive technique is immediately appreciated. Any inadequacies in placement would diminish fixation strength and may contribute to failure, underlining the absolute necessity of training and experience. Because these anchors are inserted without predrilling, tactile‐sharp skills need to be developed to confirm proper placement of the anchor with respect to depth and resistance of bone quality.

## DISCLOSURES

The author (M.B.) declares that he has no known competing financial interests or personal relationships that could have appeared to influence the work reported in this article.
